# Spatio‐temporal connectivity and host resistance influence evolutionary and epidemiological dynamics of the canola pathogen *Leptosphaeria maculans*

**DOI:** 10.1111/eva.12630

**Published:** 2018-04-17

**Authors:** Lydia Bousset, Susan J. Sprague, Peter H. Thrall, Luke G. Barrett

**Affiliations:** ^1^ CSIRO Agriculture & Food Canberra ACT Australia; ^2^ UMR1349 IGEPP INRA Le Rheu France

**Keywords:** adaptation, blackleg, *Brassica napus*, ecology, infectivity, phoma stem canker, resistance deployment strategy, virulence

## Abstract

Genetic, physiological and physical homogenization of agricultural landscapes creates ideal environments for plant pathogens to proliferate and rapidly evolve. Thus, a critical challenge in plant pathology and epidemiology is to design durable and effective strategies to protect cropping systems from damage caused by pathogens. Theoretical studies suggest that spatio‐temporal variation in the diversity and distribution of resistant hosts across agricultural landscapes may have strong effects on the epidemiology and evolutionary potential of crop pathogens. However, we lack empirical tests of spatio‐temporal deployment of host resistance to pathogens can be best used to manage disease epidemics and disrupt pathogen evolutionary dynamics in real‐world systems. In a field experiment, we simulated how differences in *Brassica napus* resistance deployment strategies and landscape connectivity influence epidemic severity and *Leptosphaeria maculans* pathogen population composition. Host plant resistance, spatio‐temporal connectivity [stubble loads], and genetic connectivity of the inoculum source [composition of canola stubble mixtures] jointly impacted epidemiology (disease severity) and pathogen evolution (population composition). Changes in population composition were consistent with directional selection for the ability to infect the host (infectivity), leading to changes in pathotype (multilocus phenotypes) and infectivity frequencies. We repeatedly observed decreases in the frequency of unnecessary infectivity, suggesting that carrying multiple infectivity genes is costly for the pathogen. From an applied perspective, our results indicate that varying resistance genes in space and time can be used to help control disease, even when resistance has already been overcome. Furthermore, our approach extends our ability to test not only for the efficacy of host varieties in a given year, but also for durability over multiple cropping seasons, given variation in the combination of resistance genes deployed.

## 1 INTRODUCTION

In modern agriculture, the implementation of genetic, technological and agronomic innovations have enabled large increases in productivity (Pretty, 2008). However, one general consequence of modernization has been simplification of the genetic, physiological and spatial elements of agricultural landscapes. Such ecological homogeneity facilitates the invasion, adaptation and proliferation of plant pathogens within agroecosystems. While a key development in terms of disease control has been the breeding and deployment of crop varieties with genetically controlled resistance to pathogens, resistance has often not proven durable because many of the pathogens that pose the greatest threats to crop yields have repeatedly evolved the ability to overcome resistance genes following their deployment (Burdon, Barrett, Rebetzke, & Thrall, 2014; Burdon, Zhan, Barrett, Papaïx, & Thrall, 2016). Newly adapted pathogen genotypes that result from random mutation or recombination can quickly increase in frequency and spread throughout the landscape. Loss of efficacy of crop resistance can lead to highly damaging and economically costly outbreaks of disease. Therefore, a critical challenge in plant pathology and epidemiology is to design and implement strategies to better protect agricultural crops from rapidly evolving pathogens. While the solutions to this problem will be multifaceted, one important but frequently neglected area of focus revolves around management strategies that explicitly manipulate the epidemiological and evolutionary trajectories of pathogenic organisms. Any strategy that reduces the size of epidemics and the transfer of inoculum between seasons should also reduce the effective size of pathogen populations, limit evolutionary potential and increase resistance durability (Bousset & Chèvre, 2012, 2013; McDonald & Linde, 2002; Zhan, Thrall, & Burdon, 2014; Zhan, Thrall, Papaïx, Xie, & Burdon, 2015).

The availability of multiple sources of disease resistance facilitates manipulation of the spatial and temporal arrangement of host resistance genes within agricultural landscapes to disrupt disease dynamics (see Burdon et al., 2016 for a review). The concept of utilizing the spatial distribution of resistance diversity to control disease epidemiology is not new (Knott, 1972; Wolfe, 1985). Numerous field scale studies show that mixtures of different resistant and susceptible varieties interfere with epidemic development (e.g., Finckh & Wolfe, 2006; Wolfe, 1985, 2000), because pathogens are restricted to susceptible hosts and resistant plants act as spore sinks and barriers to dispersal during secondary cycles within the canopy (Garrett & Mundt, 1999). Related approaches such as geographic mosaics should likewise decrease connectivity between susceptible hosts and so have analogous effects at broader spatial scales (Finckh & Wolfe, 2006; Knott, 1972). The ratio and aggregation of different resistant types can be further modified to influence connectivity and encounter rates at landscape scales (Papaïx, Touzeau, Monod, & Lannou, 2014; Rimbaud, Papaïx, Rey, Barrett, & Thrall, in press). Similarly, it is common practice to temporally rotate different varieties or crop types among fields to interrupt disease transmission cycles (Bousset & Chèvre, 2012).

Spatio‐temporal variation in the deployment of crop varieties containing different resistance genes may also influence pathogen evolutionary dynamics (Bousset & Chèvre, 2013; Burdon et al., 2016). It is well known that ongoing deployment of the same resistance gene directionally selects for infective individuals in pathogen populations (Brown, 2015; Brown & Hovmøller, 2002; Van de Wouw, Howlett, & Idnurm, 2017). Here, “infectivity” is defined as the qualitative ability to infect a resistant host. In plant pathology, infectivity is synonymous with the term “virulence.” However, we prefer to use infectivity, as virulence has different meanings in the plant pathology, parasitology and evolutionary biology literature (Sacristán & García‐Arenal, 2008). Importantly, the deployment of different resistance types through space and time can influence pathogen population genetic composition (Hovmøller, Østergård, & Munk, 1997; Papaïx et al., 2014). This suggests the possibility of designing specific deployment strategies that subject a pathogen population to selective scenarios that reduce levels of adaptation.

Where trade‐offs exist between infectivity and other traits that affect pathogen fitness (e.g., spore production; Villaréal & Lannou, 2000; Thrall & Burdon, 2003; Pariaud et al., 2009; Peyraud, Cottret, Marmiesse, Gouzy, & Genin, 2016), theory predicts that regular rotation of cultivars carrying different resistance genes will select against pathogens carrying unnecessary infectivity. However, this may at least partly depend on the initial composition of pathogen populations and whether there are gametic disequilibria (Brown, 1994; Brown & Wolfe, 1990; Hovmøller, Munk, & Østergård, 1993; Kolmer, 1993). Field studies of the response of pathogen populations when challenged with mixtures have demonstrated shifts in population composition such that pathotypes showing intermediate pathogenicity and high aggressiveness significantly increased in frequency (Chin & Wolfe, 1984; but see Huang, Kranz, & Welz, 1995). At the landscape scale, diversification in the spatial deployment of resistance genes should reduce the level of genetic connectivity between host and pathogen populations across growing seasons. This may present pathogen populations with analogous evolutionary barriers, such that the emergence of pathogens combining many infectivity alleles is prevented due to trade‐offs favouring specialist types across the landscape. Thus, a better understanding of the effects of admixture of resistance sources on pathogen evolutionary change is needed.

Effective and durable crop protection management strategies should have the dual aim of making both epidemiological and evolutionary progression as difficult as possible for the pathogen. However, predicting the relative effectiveness of different strategies and the spatial scale at which they reduce epidemic development is complicated, given dependencies on the strength of trade‐offs, spatial scale of pathogen dispersal (and other pathogen life‐history features; Barrett, Thrall, et al., 2009), the number and type of resistance genes available and variation in infectivity in the pathogen population. Theoretical studies can inform our understanding of how these factors might interact to jointly influence pathogen epidemiology and evolution. For example, Rimbaud et al. (in press) used a spatially explicit simulation model to demonstrate that with two major genes for resistance, crop mixtures and rotations, can provide efficient epidemiological control even when all resistance in the population is overcome. However, these strategies are only effective when the resistant varieties comprise a high proportion of the landscape and costs of infectivity are high.

Here, we investigate the potential to design variety management strategies at the landscape scale to manage *Leptosphaeria maculans*. This fungus causes blackleg on canola (*Brassica napus*) in Australia, North America and Europe (West, Kharbanda, Barbetti, & Fitt, 2001). It produces both sexual (pseudothecia) and asexual (pycnidia) fruiting bodies during the course of an epidemic. In Australia, ascospores formed in pseudothecia on stubble (crop debris left at the end of growing season) serve as the main source of wind‐dispersed inoculum initiating epidemics in the next growing season, while conidia discharged from pycnidia disperse locally via rain splash (Hall, 1992). Spores of either type infect plants via leaves, forming leaf spots, following which cankers develop due to systemic growth of the fungal hyphae from leaf spots to the leaf petiole through vessels, and subsequently to the stem base (Hammond, Lewis, & Musa, 1985). Resistance to blackleg comes in two forms (Delourme et al., 2006). Major gene resistance is qualitative and prevents the initial infection of leaves. This form of resistance is controlled by resistance (R) genes that work by triggering a hypersensitive response. Quantitative resistance (QR) is under the simultaneous control of multiple genes and works by slowing or minimizing pathogen growth along petioles and in the stem after infection of the leaves, resulting in reduced stem canker severity at the end of the growing season.

Resistance efficacy for blackleg has proven difficult to maintain in all major cropping regions as this pathogen has repeatedly evolved infectivity against nearly all major resistance genes released so far (Brun et al., 2010; Li, Sivasithamparam, & Barbetti, 2003; Rouxel et al., 2001; Sprague et al., 2006), sometimes with almost complete yield loss (Sprague et al., 2006). Deployment of new resistance genes to date has been largely ad hoc following discovery and introduction via breeding pathways, resulting in a predictable temporal sequence of pathogen evolutionary adaptation (Van de Wouw et al., 2017). The potential for introduction of new resistances is restricted by a limited germplasm base and the amount of time it takes to develop and evaluate cultivars for commercial use (Delourme et al., 2006). Strategies to maximize the efficacy of R genes in the field are therefore critical for effective and economically viable long‐term disease management as blackleg pressure increases due to rapid expansion and intensification of the canola industry.

Previous work has shown that spatio‐temporal and genetic connectivity among pathogen populations are important considerations with respect to the management of blackleg epidemiology and evolution. Landscape structure is important for blackleg transmission, such that increased isolation of crops (up to 500 m) from canola crops grown in the preceding year was associated with lower levels of disease (Marcroft, Sprague, Pymer, Salisbury, & Howlett, 2004), and populations of blackleg collected from hosts with different R gene combinations harbour different infectivity allele frequencies (Van de Wouw et al., 2014, 2017). Management strategies that reduce levels of spatio‐temporal and genetic connectivity in the landscape thus have potential to enhance the efficacy of R gene mediated resistance in canola via both epidemiological and evolutionary control. However, empirical studies are needed to validate theoretical predictions regarding how best to manipulate the diversity of resistance in landscapes to alter transmission and pathotype composition of populations. Experiments able to simulate contrasting distances among populations, cross‐year survival, and varying levels of inoculum admixtures from sub‐populations comprised of different plant genotypes would be of great value for testing resistance gene deployment strategies identified by models (Lô‐Pelzer et al., 2010; Hossard, Jeuffroy, Pelzer, Pinochet, & Souchère, 2013; Hossard, Gosme, Souchere, & Jeuffroy, 2015; Rimbaud et al., in press). One challenge associated with experimental approaches is to effectively identify effects at field scale that may actually occur at large scales.

In this study, we designed and implemented a one‐year field plot experiment to test whether strategies based on manipulating stubble loads and resistance deployment could be meaningfully tested at the scale of field plots. More specifically, the aims of our study were firstly to test the role that variation in spatio‐temporal and genetic connectivity plays in disease epidemiology at the landscape scale, and secondly, to test whether variation in epidemiology and host resistance influences the phenotypic and genetic composition of the pathogen population.

## 2 MATERIALS AND METHODS

### 2.1 Field experiment

A field experiment was established at the CSIRO Ginninderra Experiment Station, ACT (35°12′01″S, 149°05′04″E) on 11 April 2016. A total of 60 plots (4.2 × 4.2 m) were sown in four randomized blocks. Over five rows, each block had three uninoculated and 12 inoculated treatments that were factorial combinations of host resistance type (three varieties), inoculum load (representing spatio‐temporal connectivity: high SC, low SC), and inoculum source (representing genetic connectivity: high GC, low GC). In a real landscape, harvested fields containing stubble become the spore sources for the following cropping season. For any newly sown field, the size of the initial pathogen population depends on spatio‐temporal connectivity within the landscape: the temporal component depends on spore emission in the year following harvest; the spatial component depends on the distance between fields. We simulated spatio‐temporal connectivity by varying stubble loads: plots were seeded with 20 or 10 pieces of stubble for high SC and low SC treatments, respectively. In addition, for any new field, the composition of the initial inoculum depends on genetic connectivity within the landscape. In other words, the admixture of inoculum and the proportion of infective spores depend on the distribution of host resistance in both source and newly sown fields. If the same variety or resistance type is used as in the previous year, the pathogen source population is likely pre‐adapted to the new field. We simulated this by combining stubble from three sources, to achieve two levels of pre‐adaptation, low GC and high GC as described below.

The three canola varieties (relevant blackleg resistance genes are noted in brackets) were winter canola cv. Sensation (*Rlm4*) and spring canola cvs. Hyola575CL (*Rlm6*,* Rlm4*) and Hyola50 (*LepR1, Rlm1*). Because two of our stubble sources (described below) include *Rlm1* and the frequency of isolates infective on *Rlm1* is high among those infective on *Rlm4* (our third stubble source) in Australia (since 2012, avirulence alleles for both *AvrLm1* and *AvrLm4* are nearly absent; Van de Wouw et al., 2017), only *LepR1* was considered for Hyola50. Plots were separated by inter‐plot buffers of canola. All seeds were treated with imidacloprid insecticide as per label instructions, and the seeding rate for each cultivar was adjusted to establish 40 plants/m^2^.

To construct the inoculum treatments, stubble was collected from a blackleg monitoring trial associated with the National Canola Variety Trials located at Cootamundra, NSW, in January 2016. Given the infectivity frequencies observed in previous years at this site, the cultivars ATR‐Gem (*Rlm1*,* Rlm9*), CB‐Telfer (*Rlm4*) and Hyola450TT (*Rlm1*,* Rlm4*,* LepR1*) were selected as they represented stubble sources predicted to be adapted to Hyola575CL (*Rlm6*,* Rlm4*), Sensation (*Rlm4*) and Hyola50 (*LepR1, Rlm1*), respectively. We note that no cultivars with *RLm6* were available at this site, but increased frequency of virulence on *RLm6* has been observed following the deployment of cultivars with the *RLm1* gene (Van de Wouw et al., 2016). The severity of stem canker (measured as the % diseased area of the cross section of the crown) in these inoculum sources in the previous season was 54%, 8% and 27% for ATR‐Gem, CB‐Telfer and Hyola450TT, respectively. Once collected, stubble was matured outside on bare ground and wetted daily for 15 min from 22 March 2016 to promote formation and maturation of *L. maculans* pseudothecia.

For the two genetic connectivity levels, high GC plots were inoculated with a ratio of 4:1 adapted/nonadapted inoculum, while low GC plots were inoculated with a ratio of 1:4 adapted/nonadapted inoculum. In all cases, the nonadapted proportion of the inoculum was comprised of equal parts of the two remaining nonadapted stubbles. For the Hyola575CL cultivar, during phenotyping we discovered that a priori designated high GC stubble combinations yielded populations with frequencies of adapted spores lower than low GC. Hence, the coding of high GC and low GC in the dataset was reversed to match observed levels of pre‐adaptation. On 24 May, stubble pieces were placed randomly inside a 2 × 0.5 m quadrat in the middle of each plot. High SC inoculum load pieces were placed every 10 cm and low SC every 20 cm, with the crown placed towards the outside of the quadrat in alternate directions.

Seedling establishment counts were conducted on 24 May along 6 × 1 m sections of each row to determine the mean number of plants per m^2^. On 26 July 2016, crop cover homogeneity at the rosette stage was assessed from digital pictures of a 140 × 105 cm area on each side of where inoculum had been placed. Percentage green area was calculated with an ImageJ routine (B. Moutault and JM. Retailleau, GEVES, France, personal communication) as described hereafter. The analysis was based on RGB picture segmentation with a ColorThreshold plugin. Among the 256 nuances for each colour channel, the segmentation threshold was set to 125. As wet weather in winter and spring caused waterlogging, digital pictures of all uprooted stems were taken on 21 October following final harvest 20 October, before crown canker assessment. Pictures were scored independently by two observers on a 1 to 4 scale as follows: 1 = plants not affected, long roots; 2 = stem not swollen, roots shorter; 3 = stem swollen, scarce roots; 4 = stem swollen, no roots.

### 2.2 Disease severity assessments and sampling of resulting populations

Estimates of disease severity at the leaf spot stage (start of the epidemic) were obtained from counts of blackleg leaf spots on canola plants in 1 min from one square metre (lesions counted m^−2^ min^−1^; Bousset et al., 2016) on 15 July. For each field plot, three observers counted leaf spots on green leaves while moving at constant speed (2 m per min) sideways along the length of a delimited 0.5 × 2 m area. A manual counter was used to sum leaf spot counts, and a timer was used to standardize assessment time to 1 min.

On 18 July 2016, *L. maculans* leaf spot populations were sampled by collecting 40 diseased leaves per plot, each of which was placed between layers of absorbent paper. From each leaf, one typical lesion was excised and placed on wet absorbent paper in a Petri dish. After allowing the lesion to sporulate for 24 h at room temperature, the Petri dishes were frozen at −20°C until isolation of single pycnidia.

Stem canker severity was assessed for all cultivars on 21 October (end of the epidemic). Assessments were conducted approximately 2–3 weeks prior to maturity of the spring cultivars. Due to waterlogging, the winter cultivar was assessed at the same date as spring cultivars, noting that this was 5–6 weeks prior to its maturity. Forty plants per plot were uprooted and stem cankers were scored on a 1–12 scale as follows: 1 =  no disease, 2 = 1–10%, 3 = 11–20%, 4 = 21–30%, 5 = 31–40%, 6 = 41–50%, 7 = 51–60%, 8 = 61–70%, 9 = 71–80%, 10 = 81–90%, 11 = 91–99%, 12 = 100% of crown cross section cankered.

Following assessment of canker severity, pieces of stubble comprising the crown and upper 10 cm were bagged in bird net and matured outside on bare ground at CSIRO Crace and wetted daily throughout summer and autumn for 15 min to promote formation and maturation of *L. maculans* pseudothecia. All stubble pieces were inspected under a magnifying lens (Olympus Sz40) on 3 to 4 May by a single observer, and prevalence of pseudothecia was calculated. On 24 May, the numbers of ascospores liberated from 5‐cm stubble pieces were counted for the nine populations in the high GC/high SC treatment (3 varieties x 3 replicates). A Burkard ascospore liberator (Hirst & Stedman, 1962) was used as described previously (Marcroft, Sprague, Pymer, Salisbury, & Howlett, 2003; McCredden, Cowley, Marcroft, & Van de Wouw, 2017). In each of two chambers per population, 10 stubble pieces dipped in water for 1 min were allowed to release spores for 1 h at 12 L. min^−1^ air flow. Numbers of spores trapped on Vaseline‐coated slides were counted on the whole area facing the liberator slit (area dimensions 1.5 by 13 mm) under the magnifying lens (Olympus BH‐2, D plan 40 0,65 objective and 10x/20L ocular).

### 2.3 Fungal isolations

Isolates from both the initial inoculum and leaves infected in the experiment were tested for infectivity as described in section 2.4. In July 2016, 40 single‐ascospore isolates were collected from each of the three stubble sources used to inoculate field plots. A single fragment per stubble piece, bearing mature pseudothecia, was attached with petroleum jelly to the lid of a petri dish and suspended over water agar amended with antibiotics (ampicillin 0.1 g/L, streptomycin 0.1 g/L). Single germinating ascospores were removed under a dissection binocular lens (Zeiss stereo Lumar at ×30 to ×40 magnification), transferred onto malt agar (malt extract 20 g/L, agar 20 g/L, ampicillin 0.1 g/L, streptomycin 0.1 g/L) and grown for 10 days at 19°C. All media were autoclaved for 20 min at 120°C. After cooling, ampicillin and streptomycin concentrated solution 10% w/v in water was added to a final concentration of 0.1 g/L.

After thawing the frozen leaf lesions at room temperature, 30 single pycnidial isolates per plot were produced (one isolate per leaf spot) for three of the four experimental replicates in each treatment combination. Spores oozing from single pycnidia were collected with a sterile needle under a magnifying lens (Olympus Sz40), placed on malt‐agar Petri dishes and grown for 10 days at 19°C. For each isolate, agar plugs were then transferred to V8 agar (V8 juice 160 mL/L, agar 20 g/L, ampicillin 0.1 g/L, streptomycin 0.1 g/L) and grown for 8–10 days under near UV light (NEC triphosphor 18W FL20SSBR/18‐HG). Conidia were dislodged from plates in sterile distilled water, filtered through muslin cloth, aliquoted and stored at −20°C until tested for infectivity. To standardize spore concentrations, OD570 was measured on 200 µL aliquots of spore suspension in 96‐well plates using a BioTek Powerwave HT‐1 spectrophotometer and compared to calibrated suspensions (range 10^7^ to 10^8^ spores per mL). Mean spore concentration of calibrated solutions was calculated from 10 counts of 2 aliquots with a Malassez cell (Hirschmann Neubauer 0.0025 mm^2^, depth 0.1 mm) under a magnifying lens (Olympus BH‐2, D plan 40 0.65 objective and 10×/20L ocular).

### 2.4 Infectivity tests

Following pregermination of seeds for 48 h on wet filter paper, seedlings were sown in pasteurized potting medium (compost containing recycled soil, leaf mulch, vermiculite, peat moss, river loam, perlite, and river sand with added lime and blood and bone; steam‐pasteurized at 70°C for 45 min). Plants were grown for 9–10 days under natural light in a greenhouse at CSIRO Black Mountain (mean day temperature was 19.7°C and 16.2°C at night, recorded with a Hobo U23pro data logger). A 10 μL drop of 10^7^ conidia per mL suspension was placed on each lobe of prick‐wounded cotyledons (four inoculation sites per plant) for two plants of each of the three varieties sensation (*RLm4*), Hyola50 (*LepR1*;* RLm1*) and Hyola 575CL (*RLm6*,* RLm4*). The bench was wrapped with plastic to achieve saturating humidity for 48 h, with darkness for the first 24 h. Cotyledons were cut 13–14 days after inoculation and laid on a glass plate. Pictures were taken on a blue background and later scored for infectivity phenotype by a single observer. Two control isolates with known phenotypes D13 (*AvrLm4*,* AvrLm6*,* VirLepR1*; Marcroft, Van de Wouw, Salisbury, Potter, & Howlett, 2012) and B12 (*VirLm4*,* VirLm6*,* AvrLep1;* this study) were included in each test, so that both infective (virulent) and noninfective (avirulent) reactions were generated for each variety in each test. From the pictures, tested isolates were scored as infective as soon as one compatible reaction (typical greyish lesion) was observed and noninfective otherwise. Isolates that were noninfective on all three varieties or on the variety from which they were sampled were retested to confirm the result.

### 2.5 Data analysis

#### 2.5.1 Disease severity in relation to host resistance and spatio‐genetic connectivity

Disease severity in experimental plots was considered both at the leaf spot (*n* infected leaves) and stem canker (% of crown cankered in individual plants) stages. Generalized linear modelling was used to investigate the effects of resistance (R), spatio‐temporal connectivity (SC) and genetic connectivity (GC) on levels of disease recorded in each experimental plot. Genetic connectivity (GC) was nested within resistance type (R). In addition to the main factors, leaf spot counts were included as a covariate of interest in the analysis of stem canker data. Any spatial effects (plot) were accounted for by the randomized block design, while plant cover and waterlogging were included as potential covariates due to substantial waterlogging and un‐managed grazing (rabbits, kangaroos) during the course of the experiment. Plot was treated as a fixed effect, and interactions involving block were assumed to be zero and not included in the model (Newman, Bergelson, & Grafen, 1997). For leaf spot data, we used a GLM with a quasi‐poisson distribution of errors and a log link function, while for canker data we used a binomial distribution of errors with a logit link function. Linear contrasts were calculated on model least‐square means to determine specific differences among treatment combinations.

#### 2.5.2 Infectivity and pathotype change in relation to host resistance, spatio‐temporal and genetic connectivity

Pathogen infectivity was measured for both the ascospore (inoculum) and leaf spot (experimental epidemic) populations on host lines containing *Rlm4*,* Rlm6 *+* Rlm4* or *LepR1*. This allowed direct measurement of the frequency of infectivity on individual R genes in comparison with frequencies in the initial inoculum sources. For each pathogen individual, pathotype represented the combined infectivity response on the three host lines. Infectivity was identified as “necessary” if the matching resistance gene was present in the variety from which the isolate was obtained; otherwise, it was termed “unnecessary.” From a given host, each pathotype thus had from one to three infectivity alleles, of which one or two could be unnecessary. The number of infectivity alleles is a categorical descriptor of the number of host resistance genes overcome by a pathogen isolate.

Frequencies in ascospore populations were adjusted for individual stubble treatments to obtain an expected frequency (in the absence of any selection) for each experimental treatment. For each unique treatment combination, we tested for significant departures from expected infectivity and pathotype frequencies using chi‐square tests. More specifically, for the pathotypes, we tested whether the expected frequencies of each set of seven possible pathotypes were significantly different to the frequencies of those pathotypes in the inoculum population for each genetic treatment combination (i.e., host resistance x genetic connectivity). Inoculum load was not considered, based on preliminary analyses showing that population composition was independent of population size. Analogous analyses for infectivity were performed on individual R genes to test whether the observed frequency of infectivity on different R genes in leaf spot populations was significantly different to expected values based on frequencies in the inoculum. All chi‐square tests were conducted in R (R Core Team, 2013).

Multivariate GLM was used to investigate the effects of resistance, spatio‐temporal connectivity, and genetic connectivity on the frequency of infectivity in relation to individual R genes in each experimental plot. Analyses were performed using the R‐package mvabund (Wang, Neuman, Wright, & Warton, 2012). The response variable was binary (infective/noninfective), hence we used a binomial distribution of errors. Otherwise, the models for infectivity against each of the R genes were the same as for analyses of disease severity. To investigate the factors influencing changes in pathotype frequency, we used GLM to investigate abundance of different pathotypes in response to number of infectivity alleles (1, 2 or 3), host resistance, genetic connectivity and expected infectivity pathotype on the resident host (i.e., the combination of necessary infectivities matching the resistance gene(s) present in the host). The response variable was the “observed minus expected” abundance of each pathotype, hence we used a quasi‐poisson distribution of errors with a log link function.

## 3 RESULTS

### 3.1 Populations produced by contrasting stubble mixtures

**Figure 1:**
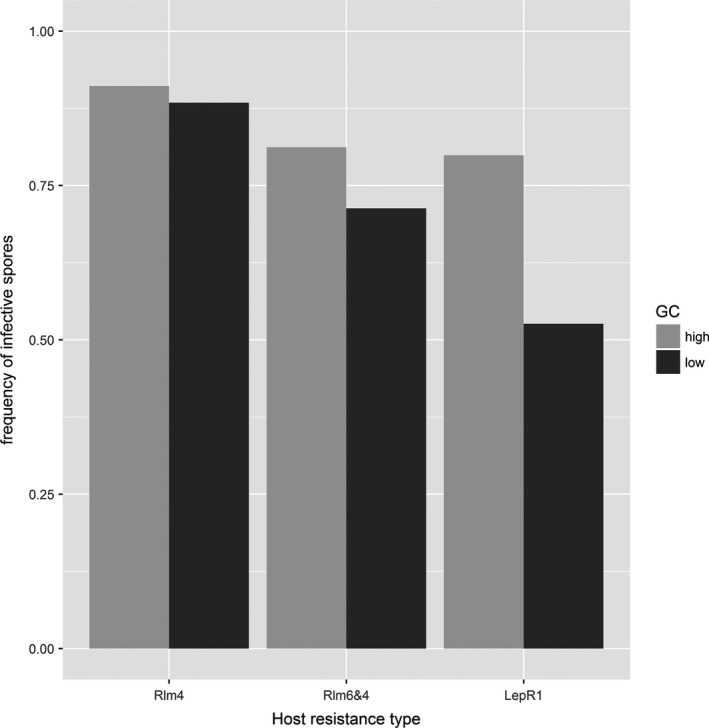
Proportion of infective ascospores in the inoculum of each treatment. These are calculated based on the pathotypes frequencies in each source population and the stubble combinations used

The proportion of infective ascospores in each treatment were calculated given the pathotypes observed in the three source stubble populations and the combinations used. These relative proportions differed among host genotypes and genetic connectivity with more infective spores in high GC than low GC for the *RLm6 *+* RLm4* and *LepR1* hosts (Figure 1).

### 3.2 Disease severity in relation to host resistance, spatio‐temporal and genetic connectivity

Disease development in interactions between canola and *L. maculans* is a two‐step process, progressing from initial infection of the leaves (as evidenced by leaf spot symptoms) to cankers of the crown over the course of the growing season. Hence, to gain a complete picture of disease dynamics, disease severity in experimental plots was assessed at both stages.

**Figure 2:**
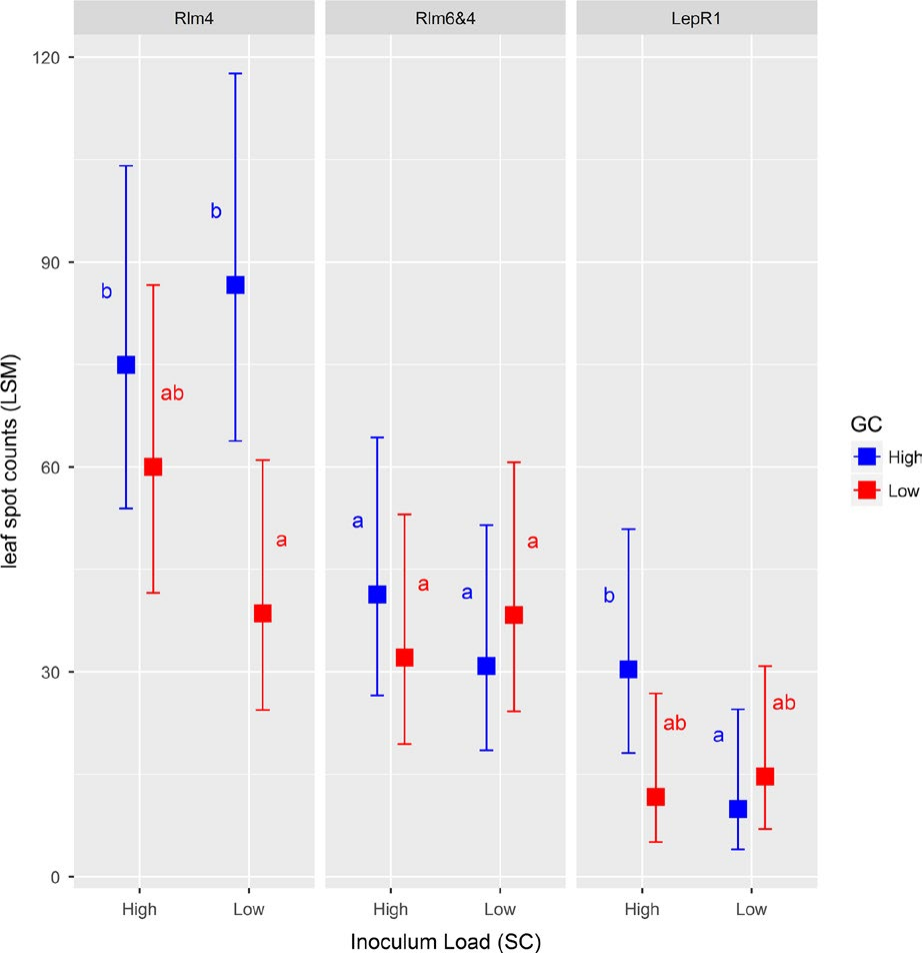
Least‐square means of leaf spot counts in experimental plots of canola for 3 different host resistance types (*Rlm4*;*RLm6 *+* Rlm4*;* LepR1*), two spatio‐temporal connectivity (SC high and low) and two inoculum genetic connectivity (GC high and low). Error bars indicate 95% confidence intervals. Within each resistance type, least‐square means sharing the same letters are not significantly different as determined by contrast tests (corrected for multiple comparisons) in *lsmeans*

At the leaf spot stage, disease severity within experimental plots was strongly influenced by all of the experimental factors and their interactions (Table 1, Figure 2). In order to simplify the final model, cover and waterlogging covariates were dropped from subsequent analyses after they proved to be nonsignificant. Effects of GC and SC differed among resistance types (Figure 2). Within plots planted with hosts carrying the *Rlm6 *+* RLm4* resistance genes, GC and SC had no effect on levels of disease. However, within experimental plots planted to hosts carrying *Rlm4* and *LepR1* resistance, GC and SC significantly influenced levels of leaf spot, albeit variably (see Figure 2 for treatment contrasts). For example, for *Rlm4* hosts, low SC resulted in less disease in low GC but not in high GC plots, whereas for *LepR1* hosts, high SC resulted in more disease on high GC plots, but did not significantly influence low GC plots.

**Table 1:** Effects of resistance type (R), spatio‐temporal connectivity (SC) and genetic connectivity (GC) on leaf spot counts within experimental plots. Genetic connectivity (GC) was nested under resistance type (R)

Tested effect	LR χ^2^	*df*	*p* value
R	26.27	2	6.37 × 10^−5^
SC	10.94	1	.002
Block	3.86	3	
GC (R)	11.52	3	.018
R × SC	12.33	2	.005
SC × GC (R)	13.82	3	.008

GLM deviance analysis. Type III tests.

**Figure 3:**
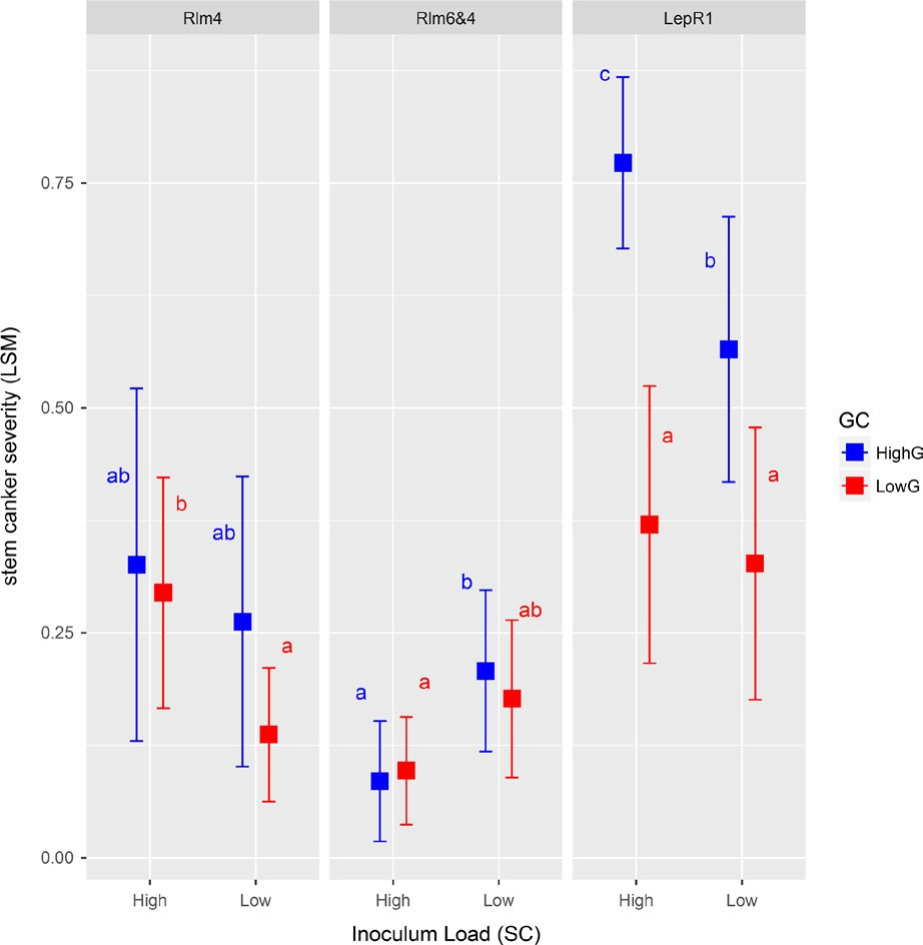
Least‐square means of canker severity in stems of plants sampled from experimental plots of canola for three different host resistance types (*Rlm4*;*RLm6 *+* Rlm4*; and *LepR1*), two spatio‐temporal connectivity (SC high and low) and two inoculum genetic connectivity (GC high and low). Error bars indicate 95% confidence intervals. Within each resistance type, least‐square means sharing the same letters are not significantly different as determined by contrast tests (corrected for multiple comparisons) in *lsmeans*

After controlling for waterlogging and ground‐cover, the progression of disease through to the stem canker stage was also influenced by all experimental factors and their interactions, although the development of stem cankers was not related to numbers of leaf spots earlier in the growing season (Table 2, Figure 3). As for leaf spots, the effects of SC and GC differed depending on host resistance. For *Rlm4* and *Rlm6 *+* RLm4* plots, significant differences in stem canker severity were only recorded for varying inoculum load in low GC treatments, but the effects of SC were in opposite directions. Specifically, for *Rlm4*, more inoculum was associated with higher levels of disease severity (as expected), but for *Rlm6 *+* RLm4*, less inoculum was associated with higher disease (Figure 3). For *LepR1*, variation in genetic composition influenced levels of disease severity (in the direction predicted) for both spatio‐temporal connectivity levels (Figure 3).

**Table 2:** Effects of resistance type (R), spatio‐temporal connectivity (SC) and genetic connectivity (GC) on stem canker severity within experimental plots. Genetic connectivity (GC) was nested under resistance type (R)

Tested effect	LR χ^2^	*df*	*p* value
R	180.05	2	2.2 × 10^−16^
SC	16.70	1	4.38 × 10^−5^
Block	12.99	3	.005
Gcover	13.41	1	.0002
Wlog	85.62	1	2.2 × 10^−16^
*N* leaf spots	0.001	1	.98
GC (R)	51.56	3	3.72 × 10^−11^
R × SC	17.02	2	.0002
SC × GC (R)	8.38	3	.039

GLM deviance analysis. Type III tests. Gcover and Wlog are covariates for ground coverage and water logging intensity. *N* leaf spots are disease severity at the leaf spot stage, estimated by the number of leaf spots counted on the plot (see text).

### 3.3 Pathotype dynamics in relation to host resistance, spatio‐temporal and genetic connectivity

**Figure 4:**
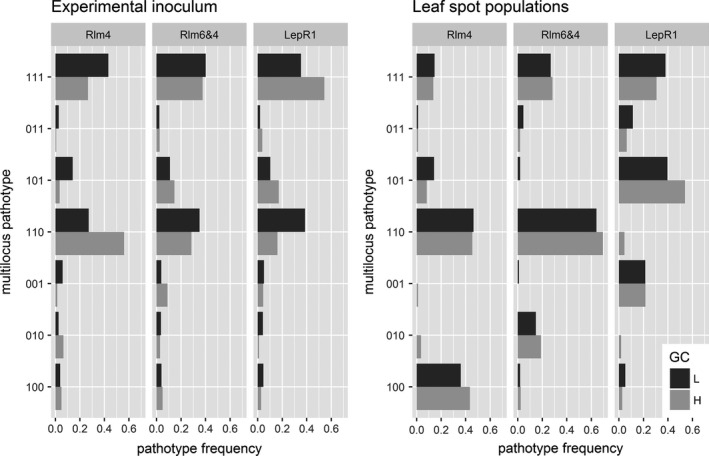
Changes in pathotype composition for each host resistance type between ascospore populations on stubble (left panel) and leaf spot populations on plants (right panel). Pathotypes are indicated for hosts with resistance genes *Rlm4*;* Rlm6 *+* Rlm4*;* LepR1*. Bars show number of times each pathotype was sampled from each stubble or plot type. Black bars are low GC, and grey bars are high GC populations. Three digit binary codes represent pathotypes (the combination of the infectivity response on *RLm4*,*RLm6 *+* RLm4* and *LepR1,* respectively) with one indicating infectivity

To investigate patterns of genetic and phenotypic change in *L. maculans* populations in response to variation in host resistance, spatio‐temporal connectivity and infectivity frequencies, we compared infectivity and pathotype frequencies in the starting inoculum with the populations recovered from our experimental treatment plots at the leaf spot stage. Frequencies in ascospore populations were adjusted for the individual stubble treatments so as to obtain an expected frequency (in the absence of any selection) for each experimental treatment. The results demonstrate significant changes in infectivity frequency across different experimental treatments (Figures 4 and S1).

For pathotypes, chi‐square tests showed significant departures from the starting pathotype frequencies in all leaf spot populations, for all 12 treatments (Table 3, Figure 4). In other words, the observed frequency of the seven possible pathotypes in the leaf spot populations was significantly different from expected, when expected frequencies were calculated based on pathotype frequencies in the source inoculum combinations (and not accounting for predicted selection by different R genes) (Table 3).

**Table 3:** Chi‐square tests for departures from expected pathotype frequencies for each experimental treatment including resistance in the host (*Rlm4*;* Rlm*6 + *Rlm4*;* LepR1*), genetic connectivity (GC) and spatio‐temporal connectivity (SC)

Resistance	GC	SC	χ^2^
*Rlm4*	High	High	142.9^a^
*Rlm4*	High	Low	280.4^a^
*Rlm4*	Low	High	215.5^a^
*Rlm4*	Low	Low	141.1^a^
*Rlm6 *+* Rlm4*	High	High	98.1^a^
*Rlm6 *+* Rlm4*	High	Low	69.4^a^
*Rlm6 *+* Rlm4*	Low	High	70.3^a^
*Rlm6 *+* Rlm4*	Low	Low	38.3^a^
*LepR1*	High	High	157.3^a^
*LepR1*	High	Low	109.9^a^
*LepR1*	Low	High	157.0^a^
*LepR1*	Low	Low	138.8^a^

Seven degrees of freedom for each comparison in seven.

a *p *<* *2 × 10^−10^.

**Figure 5:**
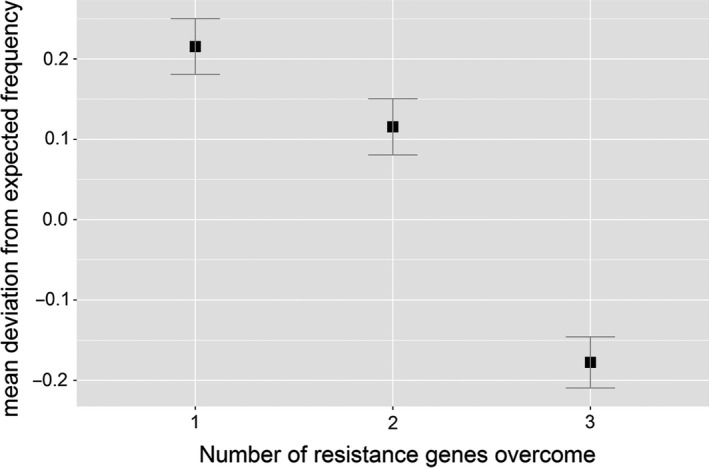
Relationship between the number of resistance genes overcome by individual pathotypes in the leaf spot populations, and the mean deviation from expected frequency based on counts of pathotypes in the starting stubble population

To evaluate which factors influenced changes in pathotype frequency (Figures 4 and S1), we used GLM to model the main effects of host resistance, genetic connectivity, infectivity and number of infectivity alleles (i.e., a categorical descriptor of the number of host resistance genes overcome by a pathogen isolate). Infectivity describes the ability of a given pathotype to infect a specific host cultivar. Noninfective strains are expected to be selected against and hence decrease in frequency (and vice versa for infective strains), while changes in pathogen frequency in relation to number of infectivity alleles may be expected if there are either costs or benefits to a pathotype carrying “unnecessary” infectivity genes. Analysis of deviance showed that both infectivity and number of infectivity alleles were significant predictors of changes in pathotype frequency, while the remaining genetic factors (host resistance type and genetic connectivity) were not significant (Table 4). Parameter estimates for the infectivity term indicate that infective pathotypes significantly increased in frequency relative to noninfective pathotypes (parameter estimate = 0.21, *t* = 7.56, *p *=* *8.5*10^−10^). The slope parameter estimate for pathotype was negative (parameter estimate = −0.16, *t* = 7.01, *p *=* *7.5*10^−11^), indicating that the departure from expected frequencies was increasingly negative when the number of infectivity alleles increased in the pathogen (Figure 5).

**Table 4:** Effect of the number of infectivity alleles (*N* alleles), resistance type (R), infectivity on the host from which the isolate was sampled (pathotype infectivity) and genetic connectivity (GC) on net changes (observed—expected) in pathotype frequency within experimental plots. Genetic connectivity (GC) was nested under resistance type (R)

Tested effect	LR χ^2^	*df*	*p* value
R	2.78	2	.25
Pathotype infectivity	49.00	1	2.6 × 10^−12^
*N* alleles	57.48	1	3.41 × 10^−14^
GC (R)	0.00	3	1.00

GLM deviance analysis. Type II tests.

**Figure 6:**
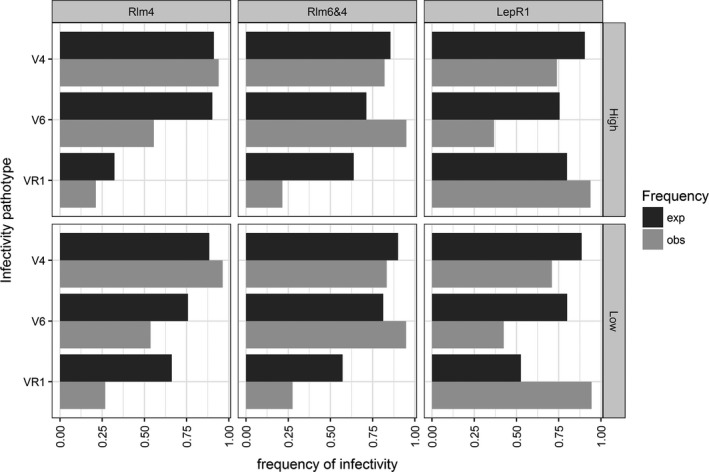
Observed (grey bars) vs. expected (black bars) frequencies of infectivity of *L. maculans* populations on different R genes (V4 on *Rlm4*; V6 on *Rlm6 *+* RLm4*; VR1 on *LepR1*) for the two genetic connectivity, high GC (upper panels) and low GC (lower panels). Columns represent populations collected from hosts with different R types (*Rlm4*;* Rlm6 *+*  Rlm4*;* LepR1*)

With regard to infectivity frequencies, we likewise observed a general increase in the frequency of infective individuals selected for by a given resistance gene, and a general decrease in the frequency of noninfective individuals when no selection was expected (Table 5, Figure 6). For example, compared to expected frequencies, the frequency of strains infective on *LepR1* increased in all *LepR1* treatments, while the frequency of strains infective on *Rlm4* and *Rlm6* decreased (Table 5, Figure 6). To test the factors that influenced changes in infectivity frequency (Figures 4 and S1), we used GLM to model the multivariate abundance of infectivity frequencies according to the main effects of host resistance, genetic connectivity, spatio‐temporal connectivity and their interactions. The multivariate analysis of deviance showed that in addition to interactions between resistance, spatio‐temporal connectivity and genetic connectivity, resistance genes were also significant predictors of infectivity frequencies, thus reinforcing the central role played by host resistance in determining the frequency of infectivity alleles (Table 6).

**Table 5:** Chi‐square tests for departures from expected infectivity frequencies for each unique experimental treatment including resistance in the host (*Rlm4*;* Rlm6 *+* Rlm4*;* LepR1*), genetic connectivity (GC) and spatio‐temporal connectivity (SC). Net pathotype frequencies (netF) are observed minus expected frequencies

Resistance	GC	SC	Locus	netF	χ2	*p* value
*Rlm4*	High	High	vir4	0.01	0.43	.51
*Rlm4*	High	Low	vir4	0.04	1.91	.17
*Rlm4*	Low	High	vir4	0.08	4.57	.03
*Rlm4*	Low	Low	vir4	0.08	5.52	.019
*Rlm6 + Rlm4*	High	High	vir4	0.02	0.35	.55
*Rlm6 + Rlm4*	High	Low	vir4	−0.12	6.42	.01
*Rlm6 + Rlm4*	Low	High	vir4	−0.09	7.94	.005
*Rlm6 + Rlm4*	Low	Low	vir4	−0.05	2.65	.10
*LepR1*	High	High	vir4	−0.12	17.64	2.67 × 10^−5^
*LepR1*	High	Low	vir4	−0.21	59.48	1.23 × 10^−14^
*LepR1*	Low	High	vir4	−0.14	18.812	1.45 × 10^−5^
*LepR1*	Low	Low	vir4	−0.22	39.56	3.17 × 10^−10^
*Rlm4*	High	High	vir6	−0.28	97.22	6.21 × 10^−23^
*Rlm4*	High	Low	vir6	−0.44	185.69	2.77 × 10^−42^
*Rlm4*	Low	High	vir6	−0.27	31.19	2.34 × 10^−8^
*Rlm4*	Low	Low	vir6	−0.18	14.62	.0001
*Rlm6 + Rlm4*	High	High	vir6	0.26	27.64	1.46 × 10^−7^
*Rlm6 + Rlm4*	High	Low	vir6	0.20	11.06	.0009
*Rlm6 + Rlm4*	Low	High	vir6	0.14	10.63	.001
*Rlm6 + Rlm4*	Low	Low	vir6	0.13	12.62	.0004
*LepR1*	High	High	vir6	−0.45	121.12	3.6 × 10^−28^
*LepR1*	High	Low	vir6	−0.33	66.62	3.3 × 10^−16^
*LepR1*	Low	High	vir6	−0.43	112.43	2.88 × 10^−26^
*LepR1*	Low	Low	vir6	−0.31	48.83	2.79 × 10^−12^
*Rlm4*	High	High	virR1	−0.11	6.05	.014
*Rlm4*	High	Low	virR1	−0.11	5.08	.02
*Rlm4*	Low	High	virR1	−0.39	53.78	2.25 × 10^−13^
*Rlm4*	Low	Low	virR1	−0.39	59.83	1.03 × 10^−14^
*Rlm6 + Rlm4*	High	High	virR1	−0.47	77.52	1.31 × 10^−18^
*Rlm6 + Rlm4*	High	Low	virR1	−0.36	31.51	1.99 × 10^−8^
*Rlm6 + Rlm4*	Low	High	virR1	−0.38	48.76	2.9 × 10^−12^
*Rlm6 + Rlm4*	Low	Low	virR1	−0.23	24.08	9.25 × 10^−7^
*LepR1*	High	High	virR1	0.18	23.64	1.16 × 10^−6^
*LepR1*	High	Low	virR1	0.09	6.32	.01
*LepR1*	Low	High	virR1	0.39	61.39	4.69 × 10^−15^
*LepR1*	Low	Low	virR1	0.45	64.69	8.78 × 10^−16^

**Table 6:** MANOVA on infectivity counts against each of the R genes (*Rlm4*;* Rlm6* and *LepR1*) depending on resistance in the host from which the isolate was collected (R), spatio‐temporal connectivity (SC), genetic connectivity (GC) and their interactions. Genetic connectivity (GC) was nested under resistance type (R)

Tested effect	*df*	MV	*Rlm4*	*Rlm6*	*LepR1*
R	2	938.5***	76.11**	292.17**	570.20**
SC	1	5.3	2.75	0.43	2.14
Block	3	12.2	3.82	3.30	5.05
GC (R)	3	16.0	2.62	6.86	6.50
R × SC	2	7.9*	2.87	1.63	3.39
SC × GC (R)	3	21.6*	5.59	5.66	10.36.

GLM deviance analysis. Type III tests. Significance: ***.001; **.01; *.05.

## 4 DISCUSSION

In this study, we used the fungal pathogen *L. maculans* to investigate how disease severity and pathogen population genetic structure vary in relation to inoculum load, levels of adaptation present in an inoculum source and host resistance. Our results support the idea that actively managing the spatio‐temporal deployment of resistance across agricultural landscapes can help control disease epidemics. In general, decreasing both spatio‐temporal (inoculum load) and genetic (inoculum pre‐adaptation) connectivity reduced disease severity, although this effect was dependent on the precise treatment combination and varied across different host resistance backgrounds. We also found evidence that the evolutionary trajectories of pathogen populations can be manipulated to reduce levels of adaptation for the following crop. Specifically, while in each host resistance treatment we observed consistent selection for infectivity, we also observed equally consistent evidence for selection against unnecessary infectivity (i.e., the ability to overcome resistance genes not present in the host).

### 4.1 Selection for and against infectivity

With regard to the evolutionary dynamics of pathogenicity, the initial pathogen populations from the three inoculum sources were differentiated for both infectivity frequencies and pathotype composition. Mixing the three sources in various proportions allowed us to inoculate host resistance types with populations with differing levels of pre‐adaptation. This approach allowed us to explicitly characterize the dynamics of adaptive change in variable *L. maculans* populations exposed to different selective pressures (host resistance genes). Previous field studies used only a single pathogen population, either exposed to different hosts (Brun et al., 2000, 2010; Delourme et al., 2014) or to contrasting stubble management practices (Daverdin et al., 2012). Our results clearly show that host resistance is the most important driver of variation in pathogen population composition (Table 3). Thus, regardless of the initial pathotype composition of the source inoculum, the outcome is largely driven by which resistance gene the pathogen population is exposed to. Our results are congruent with previous studies (Hovmøller et al., 1993), indicating directional selection for isolate‐host compatibility and predictable changes in pathotype frequencies (Figures 4 and 6). These results demonstrate that, even at the local scale of our experiment, we were able to detect the signature of adaptation to host resistance deployment, as repeatedly observed in larger‐scale cropping situations. For example, in *L. maculans*, previous studies have documented adaptation to the genes *Rlm1* in Europe and Australia (Rouxel et al., 2001; ); *LepR3* (*Rlm1*) and *LepR1* in Australia (Sprague et al., 2006; Van de Wouw et al., 2014, 2017); *Rlm7* in Europe (Balesdent et al., 2015; Leflon, 2013; Winter & Koopmann, 2016); *Rlm3* in Canada (Zhang et al., 2016).

The replicated design of our experiment enabled further analysis demonstrating that selection against unnecessary infectivity also repeatedly occurred, regardless of the composition of the initial pathogen population or the specific resistance gene in the host (Figure 4). The assumption of infectivity costs is one that has frequently been included in evolutionary models of host–pathogen interactions, particularly those that assume gene‐for‐gene scenarios (e.g., Sasaki, 2000; Thrall, Barrett, Dodds, & Burdon, 2016). This is at least partly because the inclusion of such costs provides an obvious mechanism for maintaining polymorphisms in resistance and infectivity. Likewise, costs of infectivity have been included in applied models of adaptation to host resistance (Brown, 2015; Leach, Vera Cruz, Bai, & Leung, 2001), from which they emerge as important predictors of host resistance durability, pathogen evolutionary potential and epidemiology (REX consortium, 2013). However, despite their ubiquity in theoretical frameworks, there are still relatively few empirical examples of trade‐offs between different components of pathogen fitness, particularly under field conditions.

A trade‐off between infectivity and aggressiveness (i.e., the quantitative component of the interaction, measured as spore production per pustule) leading to selection against unnecessary infectivity has been documented in a natural plant–pathogen metapopulation of the *Linum‐Melampsora* interaction (Thrall & Burdon, 2003). In addition, a number of studies have attempted to measure such costs in either the field or under controlled conditions (e.g., Chin & Wolfe, 1984; Huang et al., 2006, 2010; Barrett, Bell, Dwyer, & Bergelson, 2011; Zhan & McDonald, 2013). However, temporal changes in infectivity patterns in agricultural systems are not always consistent with theoretical expectations. In some cases, seemingly unnecessary infectivity has not shown decreases over time (Brown, 2015; Caffier, Hoffstadt, Leconte, & de Vallavieille‐Pope, 1996) or has even increased in the absence of the corresponding selection pressure, due to hitchhiking (Van de Wouw et al., 2010, 2017; Zhan, Yang, Zhu, Shang, & Newton, 2012) or contrasting use of resistance genes in winter and spring varieties (Bousset, Hovmøller, Caffier, de Vallavieille‐Pope, & Østergård, 2002; Hovmøller et al., 1997).

Critically, the efficacy of a given combination of resistance genes must be determined in the context of the composition of the local pathogen population experienced by the host (Papaïx, Monod, Goyeau, du Cheyron, & Lannou, 2011; Wu et al., 2015). In the case of *L. maculans*, methods for testing the potential efficacy of a set of host resistance combinations by sowing them into the previous years’ stubble have been developed (Marcroft et al., 2012). Our experimental design further allowed us to evaluate changes in the infectivity profile of pathogen populations (including unnecessary infectivity). This extension of the approach developed by Marcroft et al. (2012) raises the possibility of testing host resistance combinations, not only for efficacy in the following year, but also for stability over several cropping seasons. Clearly, pathogen evolutionary trajectories (i.e., responses to selection pressure) are at least partly determined by the pathotype composition and number of infectivity alleles carried by individuals in the initial pathogen population. For example, the superiority of pyramiding resistance genes over crop rotation schemes or mixtures depends on the absence of corresponding isolates carrying appropriate combinations of infectivity genes (Lof, de Vallavieille‐Pope, & van der Werf, 2017; REX consortium, 2016). While precise early characterization might be hampered by low initial frequencies of infective pathotypes, the ability to expose many local populations to different resistance combinations and compare pathogen adaptive responses may enable identification of the optimal deployment strategies for a given cropping region.

### 4.2 Spatio‐temporal connectivity

In agricultural landscapes, pathogen persistence requires effective transmission between seasonal plantings of crop hosts. Thus, changes in population composition across years depends on selection during the cropping season (e.g., selection for infectivity by host resistance genes), demographic and evolutionary dynamics associated with surviving the inter‐season (Barrett, Kniskern, Bodenhausen, & Zhang, 2009) and then transmission and infection of crop hosts at the beginning of the next season. It has been proposed that selectively reducing the contribution of pre‐adapted pathogen populations, by rotation or cultural practices, could slow evolution (Bousset & Chèvre, 2013). Currently, stubble from the previous year is the primary source for *L. maculans* inoculum (Marcroft et al., 2004) although changes in tillage practices (McCredden et al., 2017) or shorter rotations with more frequent return of canola on the same fields (Kutcher et al., 2013; Harker et al., 2015) might alter this situation. Given that the survival of *Leptosphaeria maculans* decreases over the first year following harvest, options for selectively reducing contributions of different inoculum sources could include increasing the distance to a spore source (Bousset, Jumel, Garreta, Picault, & Soubeyrand, 2015; Marcroft et al., 2004; Savage, Barbetti, MacLeod, Salam, & Renton, 2013), stubble management by burial (Huang, Fitt, & Hall, 2003; Marcroft et al., 2004; Naseri, Davidson, & Scott, 2008; Thürwächter, Garbe, & Hoppe, 1999), flooding (Cai et al., 2015) or chemical application (Wherrett, Sivasithamparam, & Barbetti, 2004). The experimental design described in this study allowed us to vary the contribution of different inoculum sources, thus facilitating tests of theoretical predictions and insights into the efficacy of different deployment strategies.

In our experiment, genetic connectivity (stubble load) did not impact pathogen population composition. However, it did influence the severity of leaf spot and cankers in the resulting epidemics (Figures 2 and 3). These results are consistent with observations that early season infections lead to canker development (Marcroft et al., 2005) and that ascospore loads impact blackleg severity (Wherrett et al., 2004). Reducing ascospore loads is desirable, because in field experiments over three cropping seasons, canola pod number and seed yield declined linearly as blackleg severity increased (Hwang et al., 2016). Benefits can thus be obtained by better management of stubble loads to reduce transmission of inoculum between cropping seasons, which depends jointly on distance between fields and the size of the source population (Marcroft et al., 2004). Transmission between fields can be predicted from spore dispersal (Bousset et al., 2015; Marcroft et al., 2004; Savage et al., 2013). Thus, spatially explicit models can be used to study and ultimately design combinations of landscapes, cultivar choice and tillage practices promoting resistance durability against blackleg (Hossard et al., 2015; Lô‐Pelzer et al., 2010).

The number of leaf spots was a poor predictor of canker severity (Figures 2 and 3). In part, this might be because we only recorded the occurrence of leaf spots at a single point in time (previous work has shown that the ability to predict canker severity may depend on at what point during an epidemic leaf spot data is collected; Powers, Pirie, Latunde‐Dada, & Fitt, 2010). Further, environmental factors might affect canker severity, for example, our analyses showed that waterlogging was a significant covariate. Contrasting levels of quantitative resistance might also alter the ability to predict canker severity from leaf spot data; however, the level and effect of quantitative resistance are difficult to assess (Delourme et al., 2006). Finally, the timing of canker scoring was chosen 2–3 weeks prior to the maturity of the spring cultivars (Hyola 50 and Hyola 575CL). Due to concerns about waterlogging, we chose to assess the winter cultivar Sensation on the same day, that is, 5–6 weeks prior to maturity which would likely have reduced canker severity estimates, given this increases over time prior to maturity.

Decrease in the size of stubble source populations over time depends on cropping practice and climate. A number of previous studies have focused on understanding and forecasting the timing of fruiting body maturation and ascospore release (Brachaczek, Kaczmarek, & Jedryczka, 2016; Dawidziuk, Kaczmarek, & Jedryczka, 2012; Guo & Fernando, 2005; Kaczmarek et al., 2016; Khangura, Speijers, Barbetti, Salam, & Diggle, 2007; Powers et al., 2010; Savage et al., 2013). However, only a few studies have related spore production to canker severity (Lô‐Pelzer, Aubertot, David, Jeuffroy, & Bousset, 2009). In our study, both the prevalence of pseudothecia and the number of spores released were positively linked with canker severity (data not shown). Additional experiments would be needed to relate disease control choices in one season to epidemiological consequences for the following season. Of particular interest in this context, would be to test for a host genotype effect on spore production, for example, genotypes with reduced spore production for high canker severity (Marcroft et al., 2004).

### 4.3 Genetic connectivity

Importantly, there is still no agreement on the best way to deploy available resistance genes, either individually or stacked in varieties (pyramids). On the one hand, diversifying selection has been proposed as a way of exploiting pathogens, for example, via disruptive evolutionary dynamics (Zhan et al., 2015). Alternatively, stacking genes or QTLs in pyramids is also advocated (Djian‐Caporalino et al., 2014; Fukuoka et al., 2015). It is worth noting that while stacking might be of interest when the corresponding infectivity profiles are absent from pathogen populations (Lof et al., 2017), this strategy does not allow the possibility of leveraging decreases in unnecessary infectivity as documented in our study. To allow such a decrease, the benefit of removing genes from varieties rather than stacking newly available ones into previous material should be incorporated in breeding strategies (Brown, 2015; Zhan et al., 2015).

Further, our experimental design could be extended over multiple cropping seasons to study evolutionary changes over several generations. For example, a mark–release–recapture experiment indicated differential selection between pathogenic and saprophytic phases in *Phaeosphaeria nodorum* on wheat plots (Sommerhalder, McDonald, Mascher, & Zhan, 2011). The interplay between selection for necessary and against unnecessary infectivity could be studied across both phases to investigate whether such trade‐offs occur in *L. maculans* and which pathogen life‐history traits are likely to be affected.

## 5 CONCLUSION

Understanding and predicting the dynamics of pathogen evolutionary change and designing effective strategies to prevent or disrupt such change require a multidisciplinary approach, incorporating population genetics, population dynamical modelling, manipulative experimentation, farming systems science and economics. The study described in this article provides one approach to begin to empirically disentangle the relative effects of spatio‐temporal and genetic connectivity on adaptive change in pathogen populations.

## CONFLICT OF INTEREST

All authors declare the absence of conflict of interests.

## DATA ARCHIVING STATEMENT

Data for this study are available at: https://zenodo.org with https://doi.org/10.5281/zenodo.1164133.
