# Supplementary material for: Spatio‐temporal connectivity and host resistance influence evolutionary and epidemiological dynamics of the canola pathogen Leptosphaeria maculans
Source: Evol Appl. 2018 Apr 17;11(8):1354–70. doi: 10.1111/eva.12630 (PMC6099830; doi:10.1111/eva.12630)
Supplement: Supplementary file 1 [file EVA-11-1354-s001.pdf]

Supplementary Materials

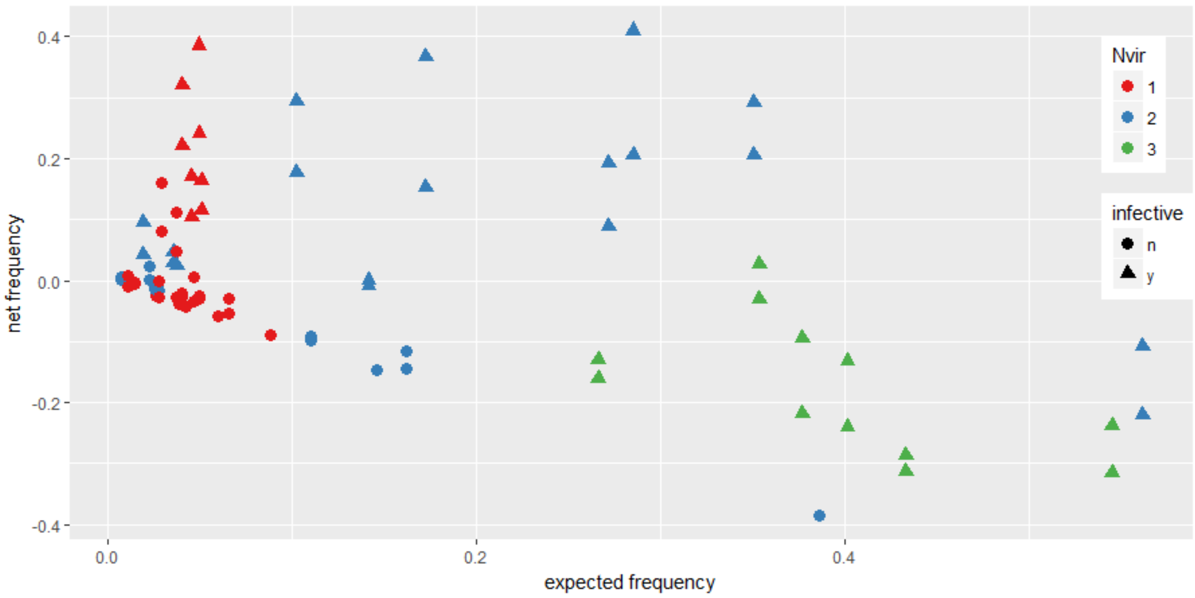

Figure S1. Selective response of pathotypes with varying infectivity profiles. Net frequency (observed minus expected) plotted against expected frequencies. Expected frequencies were calculated without considering selection imposed by resistance. Pathotypes that are non-infective on a host (colored circles) are expected to decrease in frequency, while pathotypes that are infective (colored triangles) are expected to increase in frequency. Each pathotype is colored according to its number of infectivity alleles, ranging from 1 (red) to 3 (green). Several pathotypes that are infective on the host they were isolated from had lower frequencies than predicted, most of which are infective on all three hosts.
